# Postcopulatory selection for dissimilar gametes maintains heterozygosity in the endangered North Atlantic right whale

**DOI:** 10.1002/ece3.738

**Published:** 2013-08-28

**Authors:** T R Frasier, R M Gillett, P K Hamilton, M W Brown, S D Kraus, B N White

**Affiliations:** 1 Department of Biology and Forensic Sciences Program, Saint Mary's University923 Robie Street, Halifax, Nova Scotia, B3H 3C3, Canada; 2 Natural Resources DNA Profiling and Forensic Centre, Department of Biology, Trent University2140 East Bank Drive, Peterborough, Ontario, K9J 7B8, Canada; 3 John H. Prescott Marine Laboratory, New England AquariumCentral Wharf, Boston, 02110, Massachusetts, USA

**Keywords:** Genetic incompatibility, genetic variation, mate choice, mate incompatibility, right whale

## Abstract

Although small populations are expected to lose genetic diversity through genetic drift and inbreeding, a number of mechanisms exist that could minimize this genetic decline. Examples include mate choice for unrelated mates and fertilization patterns biased toward genetically dissimilar gametes. Both processes have been widely documented, but the long-term implications have received little attention. Here, we combined over 25 years of field data with high-resolution genetic data to assess the long-term impacts of biased fertilization patterns in the endangered North Atlantic right whale. Offspring have higher levels of microsatellite heterozygosity than expected from this gene pool (effect size = 0.326, *P* < 0.011). This pattern is not due to precopulatory mate choice for genetically dissimilar mates (*P* < 0.600), but instead results from postcopulatory selection for gametes that are genetically dissimilar (effect size = 0.37, *P* < 0.003). The long-term implication is that heterozygosity has slowly increased in calves born throughout the study period, as opposed to the slight decline that was expected. Therefore, this mechanism represents a natural means through which small populations can mitigate the loss of genetic diversity over time.

## Introduction

Randomly mating closed populations are expected to lose genetic diversity over time through genetic drift and inbreeding (Fisher 1930; Wright 1931; Hartl and Clark 1997). Specifically, alleles can be lost and heterozygosity can decrease due to drift, whereas inbreeding reduces heterozygosity. However, genetic diversity plays a key role in shaping individual fitness (Coltman et al. 1999; Dunn and Byers 2008), and therefore it is not surprising that a variety of mechanisms have evolved that can result in patterns of reproduction deviating from random expectations in ways that increase the genetic diversity (and subsequent fitness) of offspring (see Jordan and Bruford 1998; Tregenza and Wedell 2000; and Kempenaers 2007 for reviews). If such mechanisms are widespread in a population, then these individual-based strategies can reduce the rate at which genetic diversity is lost from the population as a whole, and thus impact the overall extinction probability of the population (Saccheri et al. 1998; Westemeier et al. 1998; Spielman et al. 2004).

Examples of such mechanisms include both pre- and postcopulatory strategies. Precopulatory strategies (generally referred to as “mate choice”) for reducing the loss of genetic diversity typically involve mechanisms to avoid inbreeding, such as natal dispersal (Pusey and Wolf 1996; Szulkin and Sheldon 2008), active avoidance of mating with relatives (Pusey and Wolf 1996; Stow and Sunnucks 2004), or the preference of mates that are genetically dissimilar (Wedekind et al. 1995; Hoffman et al. 2007). Two primary postcopulatory mechanisms have been described, both of which have been referred to as: “genetic incompatibility.” The first involves increased rates of fetal loss when offspring are too genetically similar to the mother, which results in a breakdown in self-/non–self-recognition and subsequent fetal abortion (Ober et al. 1998). The second mechanism involves increased fertilization/pregnancy success between genetically dissimilar gametes (Birkhead et al. 2004; Evans and Marshall 2005; Dziminski et al. 2008), which presumably results from selection for heterozygous offspring (Brown 1997). For species with internal fertilization, it is generally not known if this results from prefertilization processes (e.g., cryptic sperm choice by females) or from differential mortality of zygotes (Olsson et al. 1999).

Although these mechanisms have been documented in a wide range of species, the studies have focused on short-term questions (e.g., whether or not the mechanisms are acting), and few data are available regarding the subsequent long-term/evolutionary implications. As a result, future progress in this field will rely on longer term studies of wild populations to identify the long-term implications of these processes in nature (Birkhead and Pizzari 2002; Mays and Hill 2004).

Conceptually, it seems likely that the impacts of these processes will become larger as population size decreases. In large populations there will be a large number of mates and gametes to choose from. Thus, most mates or gametes chosen at random will meet the criteria of unrelated, dissimilar, and/or compatible (whichever mechanism[s] is acting in the particular population). However, as population size declines, the number of mates or gametes that meet the criteria will also decline, resulting in mate choice/compatibility having an increasing impact on patterns of reproductive success. Consequently, the ability to detect and study such patterns should be higher for small populations. Here, we use over 25 years of data from the North Atlantic right whale (*Eubalaena glacialis*) to test hypotheses regarding the impact of mate choice and genetic incompatibility on patterns of reproductive success in this endangered species.

**Figure 1:**
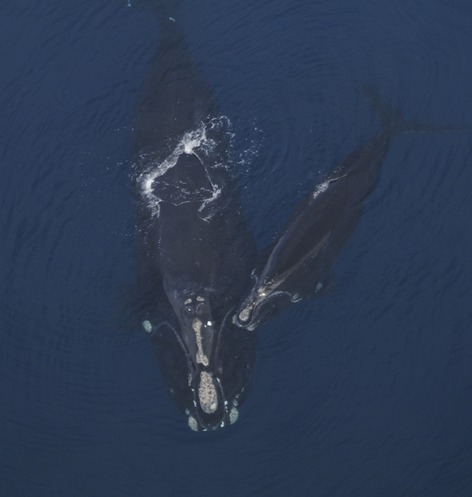
A mother and calf North Atlantic right whale (*Eubalaena glacialis*). Photo taken by Jessica Taylor/New England Aquarium under NOAA permit #655-1652-01.

The North Atlantic right whale (Fig. 1) is one of the world's most endangered large whales, with 400–500 individuals remaining (Hamilton et al. 2007; Pettis 2012). Despite over 75 years of international protection, there has been little sign of recovery (Kraus et al. 2005). The two most likely factors limiting population recovery are a high rate of anthropogenic mortality due to ship strikes and entanglement in fishing gear (Kraus et al. 2005), and a reproductive rate that is substantially lower than their known potential (Frasier et al. 2007a; Kraus et al. 2007; Browning et al. 2010).

North Atlantic right whales have among the lowest levels of genetic variability yet reported for a wild population (Frasier et al. 2007b), which has led to the hypothesis that low genetic diversity is at least partially responsible for the reduced reproductive performance. Preliminary support for this hypothesis came from restriction fragment length polymorphism (RFLP) studies, which found that band sharing between mothers and offspring was significantly lower than expected (Schaeff et al. 1997). This result suggested that mating events between genetically similar individuals are less successful, whereas those between genetically dissimilar individuals were more likely to result in successful fertilizations and pregnancies. Here, we combined data from 28 microsatellite loci with data from the major histocompatibility complex (MHC) to extend these analyses and test hypotheses regarding the influence of genetic variability on patterns reproductive success. Specifically, we tested three major hypotheses for each type of marker (microsatellites and the MHC): (1) offspring have higher levels of genetic variability than expected from random mating/fertilization within this gene pool; (2) individuals show precopulatory mate choice for genetically dissimilar mates; and (3) pregnancies or fertilizations are more successful between genetically dissimilar gametes, or when the gametes result in a fetal genotype dissimilar from that of the mother.

## Methods

### Photo-identification and maternity data

Individual North Atlantic right whales are recognized using natural markings: primarily callosity patterns on their heads, as well as variation in ventral pigmentation and scarring patterns (Kraus et al. 1986; Hamilton et al. 2007). Photo-identification research has been ongoing, almost year round, since 1980. Calves nurse for ∼1 year (Hamilton et al. 1995), and therefore mothers are identified by this long-term association with their calves (Knowlton et al. 1994). Given the extensive nature of the surveys on both the calving grounds and in spring, summer, and fall nursery and feeding areas, most calving events are identified each year (Browning et al. 2010).

### Genetic and paternity data

Small skin samples have been collected from right whales, in association with the photo-identifications, since 1988 using a crossbow equipped with a modified bolt and biopsy dart (Brown et al. 1991). DNA is extracted from each sample using standard protocols (e.g., Wang et al. 2008). Individual-specific genetic profiles are obtained for each sample based on molecular sex determination (Shaw et al. 2003), sequencing a portion of the mitochondrial control region (Malik et al. 1999), and genotype analysis at 35 microsatellite loci (Frasier et al. 2006). Although all samples are typed at 35 loci, 28 were used for subsequent analyses, with two loci being removed due to high estimates of null alleles and five being removed due to significant signs of linkage (Frasier et al. 2007a). These 28 loci provide very high resolution for discriminating between individuals and assessing relatedness. For example, the probability of identity *P*_(ID)_ (Paetkau and Strobeck 1994) is 5.92 × 10^−11^, and the *P*_(ID)sib_ (Waits et al. 2001) is 3.61 × 10^−5^. A comparison of the genetic and photo-identification data showed that error rates associated with both methods are very low (0.0308 errors/identification for the photo-ID data, and 0.00121 errors/locus for the genetic data), indicating that both provide reliable means of individualization for this species (Frasier et al. 2009). Previous analyses based on these data showed that with this large number of loci, different paternity assignment methods converge on similar assignments (Frasier et al. 2007a). As a result, paternity is assigned on an annual basis using the method of exclusion, where paternity is only assigned when all but one male is nonexcluded as a potential father, and all other males mismatch at a minimum of two loci. These paternity assignments are stored in the genetic database. Two mismatching loci were chosen as the criterion for exclusion to prevent false exclusions from potentially being made as the result of mutations or genotyping errors. Based on available data on microsatellite mutation rates (e.g., Ellegren 2004), and genotyping error rates in this study (Frasier et al. 2009), the probability of two events in one mother–father–offspring triad is exceedingly low. Based on this same logic, two mismatching loci are also the criterion for exclusion in human forensic paternity cases (e.g., Butler 2005), and therefore this criterion also seemed appropriate for our study. Frasier et al. (2007a) reported paternity assignments from 1980 to 2001, and here we have added data from years 2002–2006, as well as a reanalysis of all previous years to account for more potential fathers that have been sampled.

Although the standard method used for paternity assignment in this species is based on exclusion, in this study we also used the 95% criterion in cervus (Marshall et al. 1998). Specifically, paternity analyses were conducted independently for each year, to account for the maturation of calves from earlier years to parents in later years, and changes in the pool of candidate males each year. Critical values of △ (the ratio of the likelihood of the two most likely males as fathers) for the 95% confidence criterion were estimated based on 10,000 simulation cycles and allowing a genotyping error rate of 0.010.

All subsequent analyses were conducted using each of these data sets (i.e., once using the paternities assigned using exclusion, and a second time using the paternities assigned using the 95% criterion of cervus). Because all subsequent analyses (and interpretation) are based on the paternity assignments, this approach ensures that the results obtained were robust to different strategies of paternity assignment.

It has recently been recognized that paternity assignment methods can produced biased results, where assigned fathers may be biased toward males that are more/less heterozygous, or more/less genetically similar to the mother, depending on the assignment method used and the characteristics of the loci (Wetzel and Westneat 2009; Wang 2010). The recommended solution to this problem is to use one set of markers for paternity assignment, and a different set of markers for subsequent analyses. Preliminary tests of this approach indicated that this was not an option with our data set due to the very low levels of genetic variability in this species; hence, all loci were needed for confidence in paternity assignment. Although this recommended approach could not be taken, there are other means to assess whether or not the results obtained have been biased in this manner. We have taken steps to ensure that our results are not biased by these paternity assignment issues, and these are discussed in greater detail in the Discussion.

For this study, a 240 bp fragment of exon 2 of the class II DRβ major histocompatibility (MHC) region of all mother–father–offspring triads was also analyzed to aid our assessment of reproductive patterns (Gillett 2009). This region was chosen because it is known to influence patterns of precopulatory mate choice, as well as postcopulatory fertilization patterns and zygote survival in many vertebrates (Wedekind et al. 1995; Ober et al. 1998; Jacob et al. 2002; Milinski 2006).

### Statistical analyses

Genetic variation in offspring was quantified using two metrics, internal relatedness (IR, Amos et al. 2001) and homozygosity by loci (HL, Aparicio et al. 2006). Both of these metrics are commonly used to quantify the genetic diversity of individuals (e.g., Rijks et al. 2008) and are essentially weighted measures of heterozygosity, where the weights are based on the frequencies of the two alleles present (IR), or the allelic variability in each locus (HL). Simulations, generated using the program STORM v. 2.0 (Frasier 2008), were used to test if offspring have higher levels of heterozygosity than expected given the gene pool, as was observed with the RFLP data. Briefly, whales that had been assigned as parents represented the available gene pool. Simulated mating pairs were generated by sampling only these individuals, with replacement, to generate the same number of mating pairs as for the observed data. One offspring for each pair was then generated based on Mendelian inheritance, and IR and HL were calculated for each offspring. This process was repeated 1000 times to generate the expected distributions of these metrics for this gene pool. Calculations of effect size for all comparisons between observed and expected values were based on Cohen's *d*, *d* = (μ_1_ − μ_2_)/√[(σ_1_^2^ + σ_2_^2^)/2] (Cohen 1988), where μ and σ represent the mean and standard deviation, respectively.

An observed excess of heterozygosity in offspring could be due to three possible processes: (1) precopulatory mate choice for genetically dissimilar (or unrelated) mates; (2) genetic incompatibility based on fetal loss resulting from a breakdown in self-/non–self-recognition; and (3) genetic incompatibility based on heterozygosity.

Randomization processes were used to test if individuals show mate choice preferences for genetically dissimilar mates, where relatedness was used as a proxy for genetic similarity. The relatedness of observed mating pairs was calculated using the method of Li et al. (1993), with each locus weighted as described in Lynch and Ritland (1999). Then, individuals were randomly shuffled to create a new set of observed mating pairs, and the relatedness values for these randomized pairs were calculated. This shuffling process was repeated 1000 times to generate the distribution of expected relatedness values if individuals do not show a preference for dissimilar mates.

**Figure 2:**
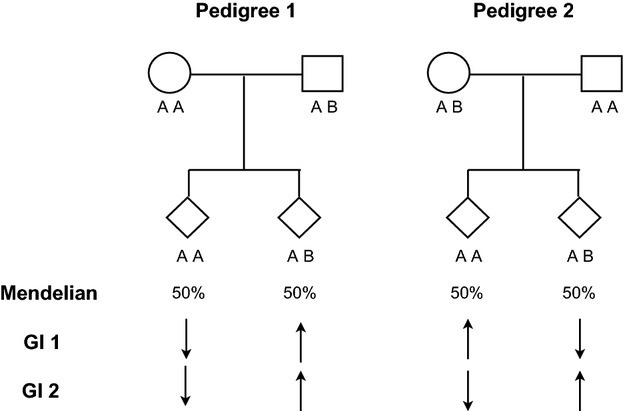
Example pedigrees showing expectations in the genotypes of surviving offspring under hypotheses of Mendelian inheritance, genetic incompatibility based on self-/non–self-recognition (GI 1), and genetic incompatibility based on heterozygosity (GI 2). Arrows indicate either an increase or decrease relative to Mendelian expectations. Pedigree 1 represents a scenario where both processes result in the same expectations, and pedigree 2 represents a scenario where the expectations are different. Again, the mechanism of GI 1 results in offspring genetically dissimilar from the mother, whereas the mechanism of GI 2 results in heterozygous offspring, regardless of their similarity to the mother.

Each of the two hypotheses of genetic incompatibility results in expectations of how allele inheritance in observed (surviving) offspring should differ from Mendelian expectations. Under the hypothesis of fetal loss based on self-/non–self-recognition (AI_FL_), observed offspring should represent those fertilizations where the zygote disproportionately inherited paternal alleles that resulted in a fetal genotype dissimilar from that of the mother. Whereas under the hypothesis of genetic incompatibility based on heterozygosity (AI_HET_), observed offspring should represent those fertilizations where the zygote disproportionately inherited paternal alleles that differed from the alleles inherited from the mother. Although these two processes of genetic incompatibility have similarities, they result in different predictions for genetic signatures in surviving offspring, as depicted in Figure 2.

Expected patterns of allele inheritance were generated by keeping observed mating pairs constant, and generating simulated offspring for each based on Mendelian inheritance. This process was repeated 1000 times to generate the distribution of expected allele inheritance values under Mendelian inheritance (e.g., without the effects of genetic incompatibility). The observed allele inheritance patterns were then compared to expected values to test if alleles were inherited in a pattern consistent with either of the two hypotheses of mate incompatibility. These analyses were conducted using the program STORM.

To assess if any observed biases in allele inheritance were due to genome-wide effects or due to a strong signal from one or a few loci, each locus was tested independently for differences between observed and expected allele inheritance patterns, and critical alpha values to delineate statistical significance were adjusted using a Bonferroni correction for multiple tests (Hochberg 1988).

To compare observed changes in heterozygosity of microsatellite loci over time with those expected without a bias in fertilization/pregnancy success, the observed mating pairs for each year were kept constant, and one offspring was generated for each based on Mendelian inheritance. This process generated the observed number of offspring for each year, from the assigned parents, but with genetic characteristics based solely on Mendelian inheritance. This process was repeated 1000 times to generate heterozygosity values expected for the offspring in each year of the study. The trend line for observed heterozygosity over time was then compared to that obtained with the simulated data to test if the observed trend is different than expected without biased fertilization/pregnancy patterns. The *P*-value was estimated based on the number of simulated data sets that had a trend line as high, or higher, than the observed data.

## Results

Paternity analyses were conducted on 180 identified mother–offspring pairs. With the exclusion method, paternities were assigned to 105 calves. All sampled males could be excluded as the father for the remaining 75 calves, and there were no unresolved paternities (cases where more than one male was nonexcluded as a potential father). Paternity assignments were similar using the 95% criterion in cervus, with 112 paternities being assigned (Table S1). There were no discrepancies in paternity assignments between the two approaches (i.e., there were no instances where a calf was assigned one male as a father with one approach, and a different male as a father using the other). Moreover, the results of all subsequent analyses were the same regardless of which paternity assignment method was used. Therefore, for clarity sake, only those results obtained using the 105 mother–offspring–father triads assigned using the exclusion approach are reported here. Analysis of the MHC was only conducted on triads identified through 2004, which represent 78 of these triads. Thus, analyses of the MHC data are based on this slightly smaller data set.

**Figure 3:**
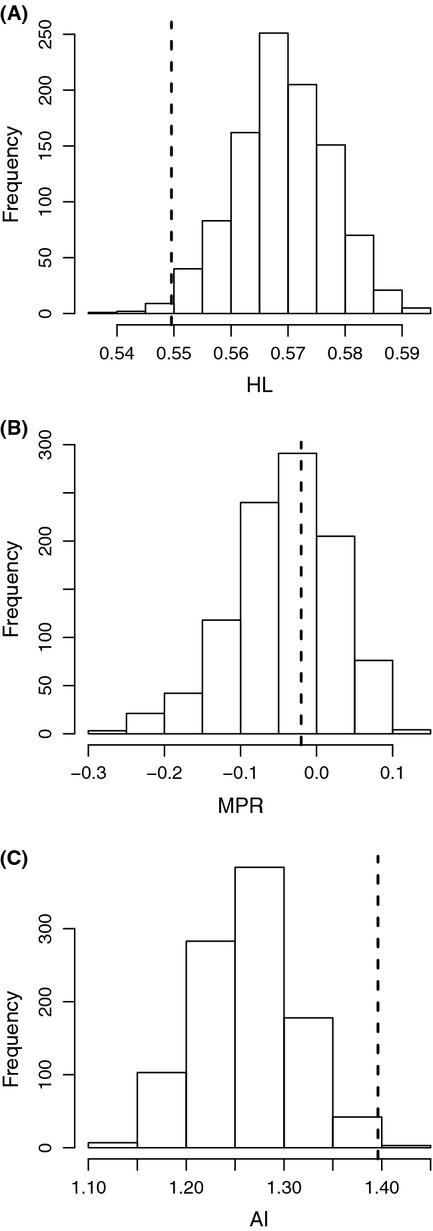
Observed and expected patterns of genetic characteristics based on microsatellites. In all cases, histograms represent the distribution of expected values and the dashed lines represent the observed values. (A) Observed and expected levels of genetic variability (HL) in the 105 offspring with both parents assigned. For the observed data, the mean and standard deviation for HL were 0.55 and 0.085, respectively. Note that HL is a measure of homozygosity, thus lower HL values correspond to higher heterozygosity. (B) Observed mating pair relatedness (MPR) and expected values if mate choice is random with respect to relatedness based on the 105 identified mating pairs, which consisted of 45 different males and 50 different females. The average relatedness between observed mating pairs was −0.02, with a standard deviation of 0.56. (C) Observed and expected allele inheritance (AI) patterns, where AI is a weighted metric quantifying the frequency at which a paternal allele is inherited that is different than that inherited from the mother (Frasier 2008).

As observed with the RFLP data, levels of genetic variability at microsatellite loci in offspring were significantly higher than expected given this gene pool, with only 10 of the 1000 simulated data sets having an average HL value as low or lower than that observed (observed mean HL = 0.549, 95% CI of expected means = 0.553–0.585, effect size = 0.326, *P* < 0.011) (Fig. 3A), and 18 having an average IR value as low or lower than that observed (observed mean IR = −0.0272, 95% CI of expected means = −0.0250–0.0412, effect size = 0.318, *P* < 0.019). This excess of heterozygous offspring does not appear to result from precopulatory mate choice for genetically dissimilar mates because the relatedness of mating pairs was not significantly lower than expected values if mate choice is random with respect to relatedness (observed mean *r* = −0.0202, 95% CI of expected means = −0.200–0.0749, *P* < 0.600) (Fig. 3B).

**Figure 4:**
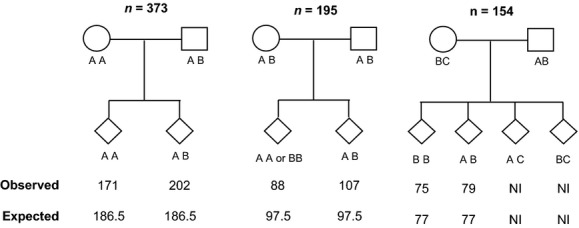
Informative pedigree structures for analyses of biased allele inheritance patterns toward heterozygous offspring for the microsatellite data. Letters do not represent actual alleles, but rather are used to designate whether or not alleles are shared. For example, there were 373 mother–offspring–father triads where the mother was homozygous and the father was heterozygous, sharing one allele with the mother. “NI” indicates triad configurations that were not informative due to the offspring inheriting a maternal allele that was not present in the father, and therefore would be heterozygous regardless of which allele was inherited from the father (i.e., there was not an opportunity for biased allele inheritance). The number of pedigrees here exceeds the number of calves with paternities assigned because each locus for a mother–father–triad can represent a different pedigree structure, with respect to allele inheritance. Thus, each triad at each locus represents one assessment of allele inheritance, for a total of (105 mother–father–offspring triads) × (28 microsatellite loci) = 2940 triad-by-locus pedigrees. The number of pedigrees shown here is less than 2940 because many pedigrees were uninformative (e.g., the mother and father were homozygous, and therefore there was not the opportunity for biased allele inheritance).

The analyses did not detect a signature of allele inheritance as expected under the hypothesis of genetic incompatibility based on self-/non–self-recognition (observed mean AI_FL_ = 1.27, 95% CI of expected means = 1.15–1.38, *P* < 0.743). However, offspring did inherit paternal alleles different than those inherited from the mother more often than expected (observed mean AI_HET_ = 1.40, 95% CI of expected means = 1.16–1.37, effect size = 0.367, *P* < 0.003), as predicted by the hypothesis of genetic incompatibility based on heterozygosity (Figs. 3C, 4). No single locus showed a significant signal of this pattern, but all showed a tendency toward the biased inheritance of a divergent paternal allele. This result suggests that the observed patterns are due to selection of divergent paternal alleles at several loci throughout the genome (i.e., a genome-wide effect), as opposed to being the result of particularly strong selection at a single locus.

At the MHC, offspring did not have significantly higher heterozygosity than expected given the gene pool (IR, *P* < 0.518; HL, *P* < 0.394) (Table S2). There was not a signal for preference for MHC dissimilar mates (*P* < 0.685), or for either biased pattern of allele inheritance (Table S2). However, previous studies in humans only found evidence for biased allele inheritance in pedigrees where the mother was heterozygous and the father was homozygous (Ober et al. 1998). When the right whale data were evaluated in this way, there was indeed a trend for offspring to inherit paternal alleles dissimilar to those inherited from the mother (Figure S1). However, the number of MHC-typed pedigrees that fit this scenario was low (*n* = 8), and therefore these analyses will be more informative in the future as more such pedigrees become available.

**Figure 5:**
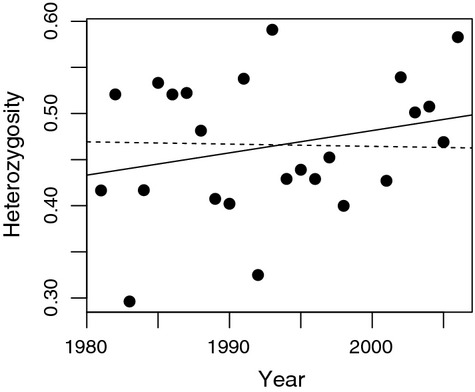
Observed (points and solid trend line) and expected (dashed line) trends in multilocus heterozygosity of calves born throughout the study period. This analysis was based on data from the 105 calves with both parents assigned, so that simulations could be conducted using the identified parents and Mendelian inheritance to generate the expected changes in heterozygosity over time if mating and fertilization patterns were random.

Heterozygosity at microsatellite loci of calves born throughout the study period showed a slight increasing trend (Fig. 5). This trend is significantly greater than would be expected if there was no selection for genetically dissimilar gametes, which would result in an expected slight decrease in heterozygosity over the same time period (observed slope = 0.00242, 95% CI of expected slopes = −0.00125–0.000763, *P* < 0.05) (Fig. 5). It is noteworthy that such a temporal trend was found given that the length of the study period is short relative to the generation time and lifespan in this species. The implication is that the true trend could be much greater than that detected, but would require a much longer study period to be adequately quantified.

## Discussion

Our results show that microsatellite heterozygosity is higher in offspring North Atlantic right whales than is expected from the gene pool, which appears to result from fertilizations and/or pregnancies being more successful between genetically dissimilar gametes. Moreover, this process has resulted in heterozygosity actually increasing in calves born throughout the 25-year study period, as opposed to the decrease expected if fertilization patterns were random.

Small populations are expected to lose genetic diversity over time through genetic drift and inbreeding: these principles form the basis of conservation genetics (Frankham et al. 2002; Allendorf and Luikart 2006). Indeed, this expected loss of genetic variability, and the subsequent decline in fitness, has been used to provide guidelines as to when populations have become too small to warrant conservation efforts due to this inevitable genetic decline (e.g., Franklin 1980). However, our results show that this process (genetic incompatibility based on heterozygosity) provides a natural means through which the loss of genetic diversity can be minimized in small populations. This important long-term implication has received surprisingly little attention.

Findings of similar biases in fertilization/pregnancy patterns are widespread (e.g., Olsson and Shine 1997; Birkhead et al. 2004). However, most studies focus on short-term implications, such as identifying whether or not a specific mechanism is acting, or how such mechanisms influence mating systems and patterns. One notable exception is a long-term study of a small population of Scandinavian wolves (*Canis lupus*). In this population, reproductive success is limited to individuals that are particularly heterozygous, which has the long-term effect of maintaining heterozygosity over generations despite an increase in the amount of inbreeding (Bensch et al. 2006). Combining the study of Bensch et al. (2006) and the results presented here shows that there is increasing evidence that the genetics of small populations is more complex than previously thought. Specifically, there are a number of widely documented natural mechanisms – such as mate choice for genetically dissimilar mates, or fertilization bias toward dissimilar gametes – that can have an increasing impact on overall patterns of genetic diversity as population size declines, that can serve to minimize the loss of genetic diversity, and the associated negative consequences, through time.

Although similar biases in fertilization/pregnancy success have been found in several other species, a mechanism for this process has not yet been identified. One primary question is whether the observed patterns are due to biased fertilization patterns (e.g., “cryptic female choice” for dissimilar sperm) or biased mortality of zygotes (Olsson et al. 1999). If it results from biased mortality of zygotes, then there should be some impact on female reproductive success: where females that lose zygotes later in pregnancy may not be able to become pregnant again during that breeding season (i.e., the later the fetus is aborted, the lower the probability of becoming pregnant again that season). It is also possible that some of this pattern could be due to differential mortality of homozygous newborn calves. Thus, it could result from processes anywhere along a continuum from differential fertilization success by dissimilar gametes, through differential mortality of homozygous zygotes throughout development, to the differential loss of homozygous newborns. Further work is needed to differentiate between these alternatives.

There is substantial evidence of fetal loss in North Atlantic right whales (Browning et al. 2010). Briefly, during the winter most pregnant females migrate to calving grounds off the coastline of Georgia and Florida. However, each year a few females who are due to reproduce migrate to the calving grounds but do not give birth. This suggests that they may have been pregnant at one time – which triggered them to migrate to the calving grounds – but that either the fetus was lost, or the calf was lost before it was detected. Using data such as these, Browning et al. (2010) estimate that the rate of fetal loss could be as high as 3 per year, which is substantial given that during this same time period the average number of calves born (for the entire species) was 14 per year. Thus, it is possible that decreased survival of zygotes from genetically similar gametes is at least partially responsible for these unsuccessful pregnancies. However, several other potential factors, such as infectious diseases and toxins, may also be influencing the rate of fetal loss (e.g., Doucette et al. 2012), and further studies are needed to elucidate the relative roles of these different factors.

The mating system of right whales represents perhaps the most extreme example of sperm competition in mammals (e.g., Frasier et al. 2007a), with males having the largest testis size-to-body size ratio of any other mammalian species (Brownell and Ralls 1986; Frasier et al. 2007a). Sperm competition results in sperm from multiple males simultaneously coexisting in the female reproductive tract, which, in addition to providing an environment for competition between sperm, is also an ideal environment for females to exercise preferences for sperm with certain traits (e.g., Olsson et al. 1996). Although the mechanism(s) through which females may be able to select the genotype of sperm are currently unknown, such mechanisms do clearly exist in some species (e.g., Carré and Sardet 1984), and likely involve genes expressed on the surface of sperm (e.g., Martin-Villa et al. 1999; Dorus et al. 2010).

From an evolutionary perspective, the cryptic female choice of dissimilar sperm would be less costly to right whale females than the mechanism of differential mortality of zygotes. Because the breeding season for this species is limited, females losing zygotes that are homozygous would reduce the likelihood of those females becoming pregnant that year. Thus, strong selection pressures would exist for earlier detection of which sperm would produce viable offspring. However, with the current data it is not possible to differentiate between these two potential mechanisms, and other data (such as the high estimated rate of fetal loss, and intense sperm competition) suggest that both mechanisms are feasible in this species.

Lastly, the question remains of how either of the hypothesized mechanisms could maintain genome-wide heterozygosity. Although mechanisms have been identified for maintaining heterozygosity at the specific loci expressed on the surface of sperm and/or at the maternal–fetal interface (Ober et al. 1998; Martin-Villa et al. 1999; Parham 2005), it is not immediately clear how they could result in the genome-wide patterns that we observed. The most likely explanation of this is what is often referred to as “associated overdominance,” where a series of genes throughout the genome are driving patterns of gametic incompatibility, and the presumably neutral markers examined show similar patterns due to linkage disequilibrium with these genes (Bierne et al. 2000; Hansson and Westerberg 2002). The theoretical rationale for this idea is that linkage disequilibrium (or, more appropriately, gametic disequilibrium) can develop throughout the genome in recently bottlenecked populations, resulting in genome-wide patterns of linkage or extremely large regions of linkage (e.g., Allendorf and Luikart 2006). For example, Reich et al. (2001) found that linkage extended to greater than 100 kb for some loci in the U.S. human population, with this large range resulting as a side effect of a recent bottleneck. Given the history of right whale exploitation, and its current small population size, it seems likely that genome-wide patterns of gametic disequilibrium could have developed, resulting in the genome-wide patterns that we observed.

Although statistically significant results were not also obtained for the MHC, a clear trend was detected in the manner expected under the hypothesis of biased allele inheritance patterns based on heterozygosity. Continuing these studies, as more pedigrees become available, will provide more adequate sample sizes for such analyses. It is interesting to note that heterozygosity was substantially higher for the MHC than for the microsatellite loci (e.g., HL = 0.397 vs. 0.550, respectively). It is widely documented that pathogens provide strong selection pressures on the MHC, often in ways that lead to heterozygote advantage, resulting in the ability to recognize and respond to a wider range of pathogens (e.g., Hedrick 1994). Thus, the already high heterozygosity at the MHC, combined with the range of selection pressures acting on it, may mask the signal of biased allele inheritance patterns at this locus. Indeed, the high heterozygosity in parents resulted in only a few pedigrees meeting the criteria where biased allele inheritance would be expected. Although the MHC is often used as a marker of choice for studies of differential mating and fertilization patterns (e.g., Penn and Potts 1999; Bos et al. 2009), our analyses clearly show that several other regions throughout the genome also likely play an important role.

It has recently been recognized that paternity assignment methods (based on both exclusion and likelihood) can result in biased paternity assignment toward males that are particularly more/less heterozygous and more/less related to the mother – depending on the allele frequency distributions – (Wetzel and Westneat 2009; Wang 2010), which can obviously lead to biases in subsequent analyses regarding mate preferences. The suggested solution is to use one subset of markers for paternity assignment and another subset for any subsequent analyses (Wetzel and Westneat 2009; Wang 2010). The low levels of genetic variation in North Atlantic right whales prevented us from using this approach because each locus is needed to obtain high resolution in the paternity analyses. However, several lines of evidence indicate that our results are not an artifact of such biases.

First, the only analyses that we conducted that could have been affected by such a bias (other than paternity itself) were our analysis of mate choice, where we tested if mates are particularly genetically dissimilar. If our exclusion-based paternity assignment was biased in the manner suggested, mothers and assigned fathers would be falsely inferred to be related (“…mothers and nonexcluded males are falsely inferred to be related when the same marker is used for both paternity and relatedness analyses, and the conclusion is true no matter which relatedness estimator is used and how allele frequencies are distributed.” [Wang 2010, p. 1903]). We found no such pattern (Fig. 3B), and the relatedness of assigned fathers to mothers had an average of ∼0. More importantly, the simulations used to test this hypothesis showed that assigned fathers were no more/less related to the mother than randomized males in the population. These data show that paternity assignments are not biased in the manner proposed.

Second, Wang (2010) showed that exclusion and likelihood paternity assignment methods can lead to opposite biases. For example, when comparing data sets of the same number of loci and allele distributions, exclusion methods tended to result in biased assignment of paternity toward males who appear *more* related to the mothers, whereas likelihood paternity assignment results in biased paternity assignment toward males who appear *less* related to mothers. We used both likelihood and exclusion paternity assignment methods, and there were no discrepancies in assignments between the two, suggesting that our assignments are not the result of such artifacts. Moreover, we observed the same results across all subsequent analyses (i.e., of mate choice and allele inheritance) regardless of which paternity assignments were used. These data indicate that our subsequent analyses are not the result of biases resulting from paternity assignment methods.

Third, our subsequent analyses were based on assessing allele inheritance patterns *given the assigned parents*. These analyses were based on comparing the observed dissimilarity of maternally and paternally inherited alleles to expected values generated by simulating offspring under Mendelian inheritance from the assigned parents. This approach means that, even if paternity was biased toward heterozygous males, it would not artificially inflate such an analysis. For example, if paternity was biased toward heterozygous males, it would be more likely for offspring to inherit differing maternal and paternal alleles, which would artificially inflate our “expected” values of allele inheritance to which we compared the observed data (which would also be similarly biased). Thus, because all analyses are based on *assigned* parents, they are essentially “standardized,” where the observed and expected data are calculated from the same paternity assignments, and would therefore be similarly affected, which serves to eliminate any potential artifacts that could result from biased paternity assignment.

**Figure 6:**
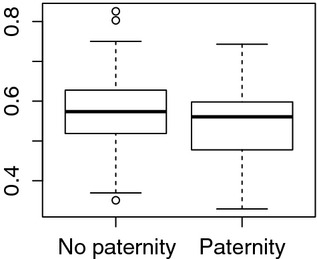
Heterozygosity values for calves with (*N* = 105) and without (*N* = 72) paternity assigned. The dark horizontal lines indicate the mean, the boxes encapsulate the middle 50% range (values in the second and third quartiles), and the whiskers delimit 1.5 times the interquartile range, which is roughly 2 SD.

Lastly, if our primary finding that offspring are more heterozygous than expected from this gene pool is an artifact of biased paternity assignments, then calves with fathers assigned should be more heterozygous than calves without fathers assigned. Figure 6 shows the data comparing heterozygosity values between calves with and without paternity assigned, and it can clearly be seen that the means and ranges of the two distributions are very similar (means are 0.315 vs. 0.299), although a larger proportion of calves with fathers assigned have *lower* heterozygosity values. As expected from this figure, there is not a statistically significant difference between the means of these two groups (*t*-test, *t* = 1.57, df = 148, *P* = 0.12).

Combined, these data show that the results and interpretations we have made are not an artifact of biases in paternity assignment methods.

## Supporting Information

**Figure S1.** Informative pedigrees for MHC data. This deviation from expected values is expected if reproduction is biased towards more heterozygous offspring. However, note the small number of MHC pedigrees that had this structure.

**Table S1.** Paternities assigned, by year, using the methods of exclusion and the 95% criterion of cervus. In both cases, paternity is assessed independently for each year to account for a changing pool of candidate males over time, as described in Frasier et al. (2007a).

**Table S2.** Observed mean values and 95% confidence intervals of expected values for metrics associated with analyses of the MHC data. Metrics include internal relatedness of calves (IR), heterozygosity-by-loci of calves (HL), mating pair relatedness (MPR), allele inheritance under the fetal loss scenario (AI_FL_), and allele inheritance under the heterozygosity scenario (AI_HET_).

## References

[b1] Allendorf FW, Luikart G (2006). Conservation and the genetics of populations.

[b2] Amos W, Worthington Wilmer J, Fullar K, Burg TM, Croxall JP, Bloch D (2001). The influence of paternal relatedness on reproductive success. Proc. R. Soc. Ser. B Biol. Sci.

[b3] Aparicio JM, Ortego J, Cordero PJ (2006). What should we weigh to estimate heterozygosity, alleles or loci?. Mol. Ecol.

[b4] Bensch S, Andrén H, Hansson B, Pedersen HC, Sand H, Sejberg D (2006). Selection for heterozygosity gives hope to a wild population of inbred wolves. PLoS One.

[b5] Bierne N, Tsitrone A, David P (2000). An inbreeding model of associated overdominance during a population bottleneck. Genetics.

[b6] Birkhead TR, Pizzari T (2002). Postcopulatory sexual selection. Nat. Rev. Genet.

[b7] Birkhead TR, Chaline N, Biggins JD, Burke T, Pizzari T (2004). Nontransitivity of paternity in a bird. Evolution.

[b8] Bos DH, Williams RN, Gopurenko D, Bulut Z, DeWoody JA (2009). Condition-dependent mate choice and a reproductive disadvantage for MHC-divergent male tiger salamanders. Mol. Ecol.

[b9] Brown JL (1997). A theory of mate choice based on heterozygosity. Behav. Ecol.

[b10] Brown MW, Kraus SD, Gaskin DE (1991). Reaction of North Atlantic right whales (*Eubalaena glacialis*) to skin biopsy sampling for genetic and pollutant analysis. Rep. Int. Whal. Commn. (Special Issue).

[b12] Brownell RL, Ralls K (1986). Potential for sperm competition in baleen whales. Rep. Int. Whal. Commn. (Special Issue).

[b13] Browning CL, Rolland RM, Kraus SD (2010). Estimated calf and perinatal mortality in western North Atlantic right whales (*Eubalaena glacialis*. Mar. Mamm. Sci.

[b14] Butler JM (2005). Forensic DNA typing: biology, technology, and genetics of STR markers.

[b15] Carré D, Sardet C (1984). Fertilization and early development in *Beroë ovata*. Devel. Biol.

[b16] Cohen J (1988). Statistical power analysis for the behavioral sciences.

[b17] Coltman DW, Pilkington JG, Smith JA, Pemberton JM (1999). Parasite-mediated selection against inbred soay sheep in a free-living, island population. Evolution.

[b18] Dorus S, Wasbrough ER, Busby J, Wilkin EC, Karr TL (2010). Sperm proteomics reveals intensified selection on mouse sperm membrane and acrosome genes. Mol. Biol. Evol.

[b19] Doucette GJ, Mikulski CM, King KL, Roth PB, Wang Z, Leandro LF (2012). Endangered North Atlantic right whales (*Eubalaena glacialis*) experience repeated, concurrent exposure to multiple environmental neurotoxins produced by marine algae. Environ. Res.

[b20] Dunn SJ, Byers JA (2008). Determinants of survival and fecundity through a population bottleneck in pronghorn (*Antilocapra americana*. J. Mammal.

[b21] Dziminski MA, Roberts JD, Simmons LW (2008). Fitness consequences of parental compatibility in the frog *Crinia georgiana*. Evolution.

[b22] Ellegren H (2004). Microsatellites: simple sequences with complex evolution. Nat. Rev. Genet.

[b23] Evans JP, Marshall DJ (2005). Male-by-female interactions influence fertilization success and mediate the benefits of polyandry in the sea urchin *Heliocidaris erythrogramma*. Evolution.

[b24] Fisher RA (1930). The genetical theory of natural selection.

[b25] Frankham R, Ballou JD, Briscoe DA (2002). Introduction to conservation genetics.

[b26] Franklin IR, Soulé ME, Wilcons BA (1980). Evolutionary change in small populations. Conservation biology: an evolutionary-ecological perspective.

[b27] Frasier TR (2008). STORM: software for testing hypotheses of relatedness and mating patterns. Mol. Ecol. Res.

[b28] Frasier TR, Rastogi T, Brown MW, Hamilton PK, Kraus SD, White BN (2006). Characterization of tetranucleotide microsatellite loci and development and validation of multiplex reactions for the study of right whale species (genus *Eubalaena*. Mol. Ecol. Notes.

[b29] Frasier TR, Hamilton PK, Brown MW, Conger LA, Knowlton AR, Marx MK (2007a). Patterns of male reproductive success in a highly promiscuous whale species: the endangered North Atlantic right whale. Mol. Ecol.

[b30] Frasier TR, McLeod BA, Gillett RM, Brown MW, White BN, Kraus SD, Rolland RM (2007b). Right whales past and present as revealed by their genes. The urban whale: North Atlantic right whales at the crossroads.

[b31] Frasier TR, Hamilton PK, Brown MW, Kraus SD, White BN (2009). Sources and rates of errors in methods of individual identification for North Atlantic right whales. J. Mammal.

[b32] Gillett RM (2009).

[b33] Hamilton PK, Marx MK, Kraus SD (1995). Weaning in North Atlantic right whales. Mar. Mamm. Sci.

[b34] Hamilton PK, Knowlton AR, Marx MK, Kraus SD, Rolland RM (2007). Right whales tell their own stories: the photo-identification catalog. The urban whale: North Atlantic right whales at the crossroads.

[b35] Hansson B, Westerberg L (2002). On the correlation between heterozygosity and fitness in natural populations. Mol. Ecol.

[b36] Hartl DL, Clark AG (1997). Principles of population genetics.

[b37] Hedrick PW (1994). Evolutionary genetics of the major histocompatibility complex. Am. Nat.

[b38] Hochberg Y (1988). A sharper Bonferroni procedure for multiple tests of significance. Biometrika.

[b39] Hoffman JI, Forcada J, Trathan PN, Amos W (2007). Female fur seals show active choice for males that are heterozygous and unrelated. Nature.

[b40] Jacob S, McClintock MK, Zelano B, Ober C (2002). Paternally inherited HLA alleles are associated with women's choice of male odor. Nat. Genet.

[b41] Jordan WC, Bruford MW (1998). New perspectives on mate choice and the MHC. Heredity.

[b42] Kempenaers B (2007). Mate choice and genetic quality: a review of the heterozygosity theory. Adv. Study Behav.

[b43] Knowlton AR, Kraus SD, Kenney RD (1994). Reproduction in North Atlantic right whales (*Eubalaena glacialis*. Can. J. Zool.

[b44] Kraus SD, Rolland RM (2007). The urban whale: North Atlantic right whales at the crossroads.

[b45] Kraus SD, Moore KE, Price CA, Crone MJ, Watkins WA, Winn HE (1986). The use of photographs to identify individual North Atlantic right whales (*Eubalaena glacialis*. Rep. Int. Whal. Commn. (Special Issue).

[b46] Kraus SD, Brown MW, Caswell H, Clark CW, Fujiwara M, Hamilton PK (2005). North Atlantic right whales in crisis. Science.

[b47] Kraus SD, Frasier RM, Pace TR, Kraus SD, Rolland RM (2007). High investment, low return: the strange case of reproduction in *Eubalaena glacialis*. The urban whale: North Atlantic right whales at the crossroads.

[b48] Li CC, Weeks DE, Charkravarti A (1993). Similarity of DNA fingerprints due to chance and relatedness. Hum. Hered.

[b49] Lynch M, Ritland K (1999). Estimation of pairwise relatedness with molecular markers. Genetics.

[b50] Malik S, Brown MW, Kraus SD, Knowlton AR, Hamilton PK, White BN (1999). Assessment of mitochondrial DNA structuring and nursery use in the North Atlantic right whale (*Eubalaena glacialis*. Can. J. Zool.

[b101] Marshall TC, Slate J, Kruuk LEB, Pemberton JM (1998). Statistical confidence for likelihood-based paternity inference in natural populations. Mol. Ecol.

[b51] Martin-Villa JM, Longás J, Arnáiz-Villena A (1999). Cyclic expression of HLA class I and II molecules on the surface of purified human spermatozoa and their control by serum inhibin B levels. Biol. Reprod.

[b52] Mays HL, Hill GE (2004). Choosing mates: good genes versus genes that are a good fit. Trends Ecol. Evol.

[b53] Milinski M (2006). The major histocompatibility complex, sexual selection, and mate choice. Annu. Rev. Ecol. Evol. Syst.

[b54] Ober C, Hyslop T, Elias S, Weitkamp LR, Hauck WW (1998). Human leukocyte antigen matching and fetal loss: results of a 10 year prospective study. Hum. Reprod.

[b55] Olsson M, Shine R (1997). Advantages of multiple matings to females: a test of the infertility hypothesis using lizards. Evolution.

[b56] Olsson M, Shine R, Madsen T, Gullberg A, Tegelström H (1996). Sperm selection by females. Nature.

[b57] Olsson M, Pagel M, Shine R, Madsen T, Dourns C, Gullberg A (1999). Sperm choice and sperm competition: suggestions for field and laboratory studies. Oikos.

[b58] Paetkau D, Strobeck C (1994). Microsatellite analysis of genetic variation in black bear populations. Mol. Ecol.

[b59] Parham P (2005). MHC class I molecules and KIRS in human history, health and survival. Nat. Rev. Immunol.

[b60] Penn DJ, Potts WK (1999). The evolution of mating preferences and major histocompatibility complex genes. Am. Nat.

[b61] Pettis H (2012). http://www.narwc.org/pdf/2012_Report_Card.pdf.

[b62] Pusey A, Wolf M (1996). Inbreeding avoidance in animals. Trends Ecol. Evol.

[b63] Reich DE, Cargill M, Bolk S, Ireland J, Sabeti PC, Richter DJ (2001). Linkage disequilibrium in the human genome. Nature.

[b64] Rijks JM, Hoffman JI, Kuiken T, Osterhaus ADME, Amos W (2008). Heterozygosity and lungworm burden in harbour seals (*Phoca vitulina*. Heredity.

[b65] Saccheri I, Kuussaari M, Kankare M, Vikman P, Fortelius W, Hanski I (1998). Inbreeding and extinction in a butterfly metapopulation. Nature.

[b66] Schaeff CM, Kraus SD, Brown MW, Perkins JS, Payne R, White BN (1997). Comparison of genetic variability of North and South Atlantic right whales (*Eubalaena*), using DNA fingerprinting. Can. J. Zool.

[b67] Shaw CN, Wilson PJ, White BN (2003). A reliable molecular method of gender determination for mammals. J. Mammal.

[b68] Spielman D, Brook BW, Frankham R (2004). Most species are not driven to extinction before genetic factors impact them. Proc. Natl Acad. Sci. USA.

[b69] Stow AJ, Sunnucks P (2004). Inbreeding avoidance in Cunningham's skinks (*Egernia cunninghami*) in natural and fragmented habitat. Mol. Ecol.

[b70] Szulkin M, Sheldon BC (2008). Dispersal as a means of inbreeding avoidance in a wild bird population. Proc. R. Soc. Ser. B Biol. Sci.

[b71] Tregenza T, Wedell N (2000). Genetic compatibility, mate choice and patterns of parentage. Mol. Ecol.

[b72] Waits LP, Luikart G, Taberlet P (2001). Estimating the probability of identity among genotypes in natural populations: cautions and guidelines. Mol. Ecol.

[b73] Wang J (2010). Do marker-based paternity assignments favour heterozygous and unrelated males?. Mol. Ecol.

[b74] Wang JY, Frasier TR, Yang SC, White BN (2008). Detecting recent speciation events: the case of the finless porpoise (genus *Neophocaena*. Heredity.

[b75] Wedekind C, Seebeck T, Bettens F, Paepke AJ (1995). MHC-dependent mate preferences in humans. Proc. R. Soc. Ser. B Biol. Sci.

[b76] Westemeier RL, Brawn JD, Simpson SA, Esker TL, Jansen RW, Walk JW (1998). Tracking the long-term decline and recovery of an isolated population. Science.

[b77] Wetzel A, Westneat DF (2009). Heterozygosity and extra-pair paternity: biased tests result from the use of shared markers. Mol. Ecol.

[b78] Wright S (1931). Evolution in Mendelian populations. Genetics.

