# Supplementary material for: Postcopulatory selection for dissimilar gametes maintains heterozygosity in the endangered North Atlantic right whale
Source: Ecol Evol. 2013 Aug 28;3(10):3483–94. doi: 10.1002/ece3.738 (PMC3797493; doi:10.1002/ece3.738)

**Supplementary** **Table 1.** Paternities assigned, by year, using the methods of exclusion and the 95% criterion of cervus. In both cases, paternity is assessed independently for each year to account for a changing pool of candidate males over time, as described in Frasier et al. (2007a).

**# of Sampled # of Fathers Assigned**

**Year Mother-calf pairs Exclusion 95% cervus**

1981 3 1 1

1982 7 4 3

1983 3 1 1

1984 8 4 4

1985 3 2 2

1986 4 3 3

1987 7 5 5

1988 3 2 2

1989 12 6 8

1990 5 2 1

1991 8 6 6

1992 7 2 3

1993 2 1 1

1994 4 2 2

1995 3 2 2

1996 11 7 8

1997 9 6 6

1998 3 2 2

1999 0 0 0

2000 0 0 0

2001 20 10 13

2002 8 4 3

2003 13 8 10

2004 13 11 9

2005 15 10 10

2006 7 4 7

**Total 178 105 112**

**Supplementary Table 2.** Observed mean values and 95% confidence intervals of expected values for metrics associated with analyses of the MHC data. Metrics include internal relatedness of calves (*IR*), heterozygosity-by-loci of calves (*HL*), mating pair relatedness (*MPR*), allele inheritance under the fetal loss scenario (*AI_FL_*), and allele inheritance under the heterozygosity scenario (*AI_HET_*).

**Metric Mean 95% CI**

*IR* 0.126 0.114 – 0.129

*HL*  0.379 0.374 – 0.383

*MPR* -0.00840 -0.0166 – -0.000234

*AI_FL_* 0.869 0.857 – 0.881

*AI_HET_* 0.825 0.813 – 0.837

**Supplementary Figure 1.** Informative pedigrees for MHC data. This deviation from expected values is expected if reproduction is biased towards more heterozygous offspring. However, note the small number of MHC pedigrees that had this structure.


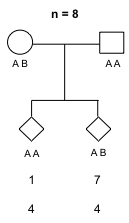

Supplement: Supplementary file 1 [file ece30003-3483-SD1.docx]
